# Enhancing Neurosurgical Education: Lessons from the COVID-19 Pandemic

**DOI:** 10.1017/cjn.2020.276

**Published:** 2021-01-05

**Authors:** Shannon Hart, Nardin Samuel

**Affiliations:** Faculty of Medicine, Memorial University, Newfoundland and Labrador, Canada; Division of Neurosurgery, Department of Surgery, University of Toronto, Toronto, Ontario, Canada

**Keywords:** Neurosurgery education, Neurosurgery residency, Virtual learning, Online

## Introduction

Interactive teaching sessions during the course of neurosurgical training are integral to resident education. With the emergence of the coronavirus disease 2019 (COVID-19) pandemic and resultant social distancing measures imposed, the medical community was forced to swiftly adapt from in-person to alternate educational methods. Reductions in clinical schedules and cancellation of regular didactic teaching sessions have raised the concern regarding maintaining adequate learning. The medical community in general, and the neurosurgical community in particular, has seen an extraordinary response to education barriers during the pandemic through the widespread introduction and use of various live and pre-recorded online educational platforms. The pandemic provides a unique time to re-evaluate educational models and improve current approach to neurosurgical education in an era where in-person learning is limited. This article examines the surge of online educational materials in the field of neurosurgery and proposes strategies for enhancing neurosurgical education in the current pandemic and beyond.

## Initiatives through Neurosurgical Organizations and Academic Institutions

**Figure 1:**
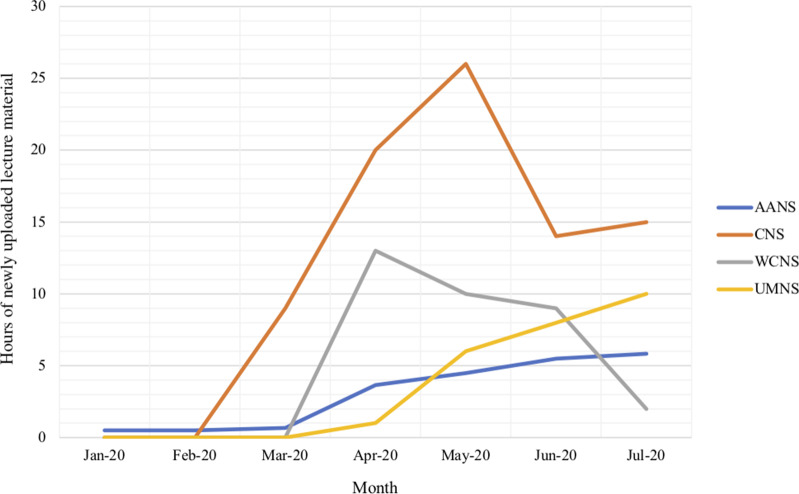
Hours of newly uploaded neurosurgical lecture material available free of charge by select organizations between January 1, 2020 and July 31, 2020. Abbreviations: CNS – Congress of Neurological Surgeons; AANS – American Association of Neurological Surgeons; WCNS – Weill Cornell Neurological Surgery; UMNS – University of Miami Neurological Surgery.

An abundance of virtual resources in the form of video conferences, poster presentations, lectures, virtual operative procedures, and “Zoomposiums^1^” have burgeoned. These initiatives include a broad range of topics from foundational aspects of neurosurgery to expert discussions on the nuances of clinical decision-making and even practical advice for residents and medical students preparing for their future neurosurgery careers. While some of these resources were made available prior to the pandemic, there has been an unequivocal surge in recent months, which can be attributed to a need for continued education in the face of meeting restrictions (Figure 1).

Both the Congress of Neurological Surgeons (CNS) and the American Association of Neurological Surgeons (AANS) have lead new initiatives created to mitigate disruptions in neurosurgical education.^2^ CNS-led initiatives include Virtual Grand Rounds and Virtual Visiting Professor, which are both weekly webinar-based lectures given by distinguished faculty, as well as the Town Hall Xperience, a weekly panelist-type discussion on practical considerations for residents and medical students, including career planning and advice for residency. The aforementioned initiatives typically use video conferencing platforms, which emphasizes an interactive learning environment. In addition to these new measures, the CNS continues to provide and promote their previously available online resources, including CNS Nexus, a case-based video library exhibiting surgical procedures; Self-Assessment Neurosurgery (SANS) Live, exams and modules to target knowledge gaps; and the Journal Club Podcast, a podcast aimed to replicate journal club sessions by discussing recent publications in neurosurgery.^2^

Similarly, the AANS announced a COVID-19 webinar series in April 2020 to address questions and issues arising in neurosurgery due to the pandemic. A more recent development called the “AANS Virtual 2020” encompasses a variety of modalities to seemingly replicate a conference as well as provide educational tools.^3,4^ The AANS Virtual 2020 includes video presentations of scientific abstracts, lecture series on controversial and/or difficult topics, over 1800 virtual poster presentations, plus multiple online courses and a virtual career fair.^3^ Most of these offerings are free to residents and students who are members of the AANS.

Individual university programs have also developed educational series that are readily accessible. The Virtual Global Spine Conference held by Weill Cornell Neurological Surgery is a weekly webinar series initiated on April 2, 2020 and has grown to have over 1200 weekly participants in just a few months.^5^ Cornell also developed a virtual Grand Rounds series offered live via Zoom and subsequently archived on YouTube.^6^ The Arkansas Neuroscience Institute, who is known for their live courses, transitioned to an online international webinar series upon the start of the pandemic, which has become substantially well attended, with over 6000 in attendance at a recent lecture.^7^ The University of Miami has rolled out similar series including their weekly Global Brain Tumor and Cerebrovascular and Skull Base Symposiums.^1^ On top of these excellent resources, many institutions have made their departmental Grand Rounds public, including Johns Hopkins,^8^ Yale School of Medicine,^9^ and University of Utah School of Medicine.^10^

A resource that precedes the pandemic but has proved to be extremely valuable in this era is the *Neurosurgical Atlas*, developed by Dr. Aaron Cohen-Gadol in 2016.^11^ Used by residents worldwide, the *Atlas* is a multimedia collection focused on describing operative techniques and neuroanatomy.^11^ In the absence of operating room (OR) experience, the *Atlas* provides virtual exposure to a large number of procedures to aid in the education of primarily medical students and residents. The *Atlas* has a sizeable group of international subscribers (over 36,000) and has seen a substantial increase in site traffic since the onset of the COVID-19 pandemic.^12^ The *Atlas* provides a supplement to live surgical experience and is particularly useful when operating time is decreased. A similar supplement that was implemented directly in response to the interruptions due to the COVID-19 pandemic is *COVIDeos-10: Survival Kit for Neurosurgical Quarantine*, a collective of operative videos accompanied by case presentations developed by Dr. Michael Lawton through the Barrow Neurosurgical Institute.^13^ Seattle Science Foundation TV is yet another initiative of the same sort, which exhibits content from hundreds of experts in a variety of medical fields, including neurosurgery.^14^ Content includes anatomical dissections, surgical demonstrations, and virtual courses. These videos acknowledge the detriment to neurosurgical education caused by the pandemic and aimed to provide regular exposure to surgeries so as to help maintain surgical technique learned thus far.

## Resources for Medical Students Applying for Neurosurgical Residency

COVID-19 has proven to be a formidable challenge for medical students planning to pursue neurosurgery in the North American 2020–2021 residency match. Typically, students in their fourth year of medical studies would complete rotations at various different programs (“sub-internships” in the US and “visiting electives” in Canada). This would allow students to have an intensive experience of each program they were interested in applying to, and likewise for faculty and residents to get to know them. These rotations are an integral part of students’ residency application and overall medical school experience. However, in light of the pandemic, in-person visiting rotations in the US and Canada were cancelled for the 2020–2021 cycle. As such, programs are now faced with the challenge of ranking applicants for residency positions, many of whom they have not had the opportunity to work with or meet.

In an effort to mitigate this barrier in the residency matching process, programs across the US have announced “virtual sub-internships” to take the place of in-person experience to facilitate a continued learning environment and familiarize applicants with the program.^15^ These externships involve a combination of web-based didactic lectures, skills sessions, and virtual surgery viewings.^16^ Furthermore, many programs have held “meet and greet” conferences between program directors, residents, and applicants to account for the socialization aspect of in-person rotations. Canadian schools are offering similar opportunities, including virtual attendance of grand rounds and meetings with the program director and residents. In addition to these sub-internships, lecture series mentioned previously in this article have included informative sessions about applying to neurosurgery in order to assist medical students in navigating the residency match. The impact or effectiveness of such approaches is yet to be determined and a thorough review of the first match cycle in the era of COVID-19 will likely inform strategies to improve and refine the sub-internship experience in the future.

## Discussion

The abundance of virtual resources developed during the COVID-19 pandemic pose an important consideration for the role of online learning in future education. As there are advantages and disadvantages of both in-person and online methods, the increasing use and adoption of virtual methods heralds a combination of the two being an ideal option.

Aside from the obvious benefit of replacing educational sessions interrupted by the pandemic, these initiatives have several advantages. For example, having access to the abundance of online lectures and study resources provides learners with a wider range of study material and allows easier information sharing worldwide. These sessions can typically be attended from anywhere, which is convenient for learners with young children or other responsibilities that may require them to be at home. Evidence shows that virtual surgical education is effective in improving knowledge and some studies even suggest superiority over in-person teaching.^17,18^ Furthermore, if constraints such as the aforementioned family responsibilities or time zone differences prohibit the attendance of live sessions, many lectures are subsequently archived online, allowing for review of the session footage at any time. Virtual webinar technology has also allowed live sessions to be interactive, so questions and discussion from the audience is enabled and encouraged. This further imitates in-person teaching sessions and conference discussions. The sessions allow learners to hear from a variety of perspectives and faculty that they otherwise would not have the opportunity to learn from, thus enhancing their overall knowledge on various topics. Finally, virtual conferences allow easier balancing of home and work life and expand access for those concerned about the financial burden of travel and accommodations. The cost reduction may be particularly beneficial for low- and middle-income countries. Overall conference attendance may increase as a result of addressing these and other barriers to attendance.

Virtual learning is undoubtedly not a substitute for in-person learning and there are some disadvantages that accompany it. As mentioned previously, the loss of live surgical experience cannot be adequately replaced in a virtual format. Operative demonstrations may supplement learning, but there is no true substitute for hands-on practice in the OR and determination of surgical competency. While no virtual experience would be able to replicate this, in dire circumstances such as a pandemic when in-person contact must be limited, it is possible that one-on-one cadaveric dissection, surgical skill practice using simulated models, or virtual reality systems may be helpful in rehearsing skills and obtaining feedback. It must be recognized that this would not be appropriate to completely replace live operating experience and may only be used as a supplement. Additionally, it can be argued that this suboptimal replacement may not be well received by learners who have had their live OR time reduced. While efforts to mitigate the detriment to education would be appreciated, it can be presumed that surgical residents would certainly miss the authentic OR experience.

Virtual learning aids may also vary in usefulness with level of training. While junior learners including medical students and junior residents may find lecture series and surgical videos incredibly useful for studying, senior learners and faculty members likely would not benefit greatly from this. That being said, international symposiums and virtual conferences would be beneficial to the latter as it would allow sharing of perspectives across the globe and discussion of recent developments in practice despite limitations in international travel. In this sense, virtual opportunities for both beginning and continuing education during the pandemic have been established. Further, there is evidence that proves virtual methods are just as effective as live conferences in terms of didactic teaching.^19^ A proposed downside of these virtual conferences, however, is that the informal networking and organic discussions that are a key attraction of conferences are difficult to replicate online.^20^

The pandemic also highlights the need for individual programs to develop a plan in the case of clinical disruption. While outside learning sources are indeed helpful, it should be the responsibility of each training center to devise how they will ensure the continuation of effective training when in-person learning is reduced. This may be an implication for future accreditation requirements, such that programs must have a documented plan for maintaining adequate education in the context of extreme circumstances.

The COVID-19 pandemic has made it evident that virtual education is a useful alternative and with current technology can provide effective learning opportunities. The pandemic has laid the groundwork for innovation and creativity in virtual neurosurgical education. The future of neurosurgical education will likely be strongly influenced by virtual learning which will serve as a useful and feasible complement to in-person education. Moving forward, the neurosurgical community can use this evolution of educational events imposed by the pandemic to implement strategies to enhance knowledge sharing and connections and to combat barriers to education worldwide.
